# Supplementary material for: Identification and validation of the common pathogenesis and hub biomarkers in Papillary thyroid carcinoma complicated by rheumatoid arthritis
Source: PLoS One. 2025 Mar 10;20(3):e0317369. doi: 10.1371/journal.pone.0317369 (PMC11892850; doi:10.1371/journal.pone.0317369)
Supplement: S1 Table — (DOCX) [file pone.0317369.s001.docx]

Table S1. PTA and RA Related Clinical Study Summary

| **Article Title** | **Country** | **Key Findings** | **Significance** |
| --- | --- | --- | --- |
| Association Between Eight Autoimmune Diseases and Thyroid Cancer: A Nationwide Cohort Study | South Korea | Compared to the general population without autoimmune diseases, individuals with one of the eight autoimmune diseases (including RA) have a significantly increased risk of thyroid cancer, especially RA patients, with a 76% increased risk. | Enhances understanding of the association between RA and thyroid cancer. |
| The five major autoimmune diseases increase the risk of cancer: epidemiological data from a large-scale cohort study in China | China | Patients with the five major autoimmune diseases in China, including RA, have an increased overall risk of cancer. | Provides epidemiological data on the association between autoimmune diseases and cancer risk. |
| Potential Cancer Risk in Patients with Rheumatoid Arthritis: A Longitudinal Korean Population-Based Analysis | South Korea | This longitudinal study in South Korea analyzed the potential cancer risks in RA patients, finding an increased incidence of certain types of cancer among RA patients, but the specific types were not detailed. | Provides a longitudinal analysis of cancer risks in RA patients. |
| Cancer risk in hospitalized rheumatoid arthritis patients | Sweden | This study focused on the cancer risk in hospitalized RA patients, finding that hospitalized RA patients have a higher cancer incidence rate than the general population, but it did not specify which types of cancer. | Focuses on the cancer risk in hospitalized RA patients. |
| The Risk of Malignancy in Korean Patients with Rheumatoid Arthritis | South Korea | This study in South Korea analyzed the risk of malignancy in RA patients, finding an increased incidence of certain malignancies among RA patients, but it did not specify which malignancies. | Provides data on the risk of malignancy in RA patients in South Korea. |
| Prevalence, incidence, and risk factors of malignancy in patients with rheumatoid arthritis: a nationwide cohort study from Korea | South Korea | This nationwide cohort study from South Korea provided data on the prevalence, incidence, and risk factors of malignancy in RA patients, finding an increased incidence of certain malignancies among RA patients, but it did not specify which malignancies. | Provides epidemiological data on malignancy in RA patients in South Korea. |
| patients | Japan | This study in Japan provided data on the cancer risk in RA patients, finding an increased incidence of certain cancers among RA patients, but it did not specify which cancers. | Provides data on cancer risk in RA patients in Japan. |
