# Supplementary material for: Identification and validation of the common pathogenesis and hub biomarkers in Papillary thyroid carcinoma complicated by rheumatoid arthritis
Source: PLoS One. 2025 Mar 10;20(3):e0317369. doi: 10.1371/journal.pone.0317369 (PMC11892850; doi:10.1371/journal.pone.0317369)
Supplement: Attachment — Submitted filename: Response to Reviewers.docx [file pone.0317369.s002.docx]

Dear reviewers, thank you for your careful review and constructive suggestions regarding our manuscript. We have revised the manuscript in accordance with the comments and marked all the amends on our revised manuscript. And point-by-point responses to the comments were as follows:

Reviewer #1: 1. Can you provide detailed steps and parameters used in your bioinformatics analyses to enhance the reproducibility of your study?

Thank you very much for your suggestion. We will put the detailed procedure and code data of the experiment in the corresponding file for other researchers to use.

2. What evaluation metrics did you use to validate the robustness and reliability of the tools and methods employed in your analyses?

We processed the data in strict accordance with the procedures, and used the DESeq2 package for differential analysis to obtain the list of differentially expressed genes as the basis of our research. Among them, the DESeq2 package is a well-known method with high false negative (actual difference, no difference in results) in common analysis. In order to avoid the chance of the differential genes obtained in the study, multiple data sets were selected for analysis, and the intersection among them was taken as the final result. Then we constructed the PPI network of differential gene set and conducted network analysis to obtain the hub gene. In order to evaluate the robustness of the hub gene we searched for, we searched for new data sets as validation to verify the results we found, and the findings were all significant. Secondly, we searched for transcription-related genes of hub gene and explored the expression differences of transcription-related genes. The results of transcription-related genes further proved the stability of our method. In this process, we carried out functional enrichment for biological exploration, and combined biology and data analysis to further explain the situation of gene changes, and the results also showed that our research was meaningful. After that, we conducted validation in clinical samples, and selected pathological specimens of patients with thyroid papillary carcinoma and rheumatoid arthritis for immunohistochemical analysis, and the immunohistochemical results also verified the results of our biogenic analysis.

3. How do your findings compare with similar studies in this field? Can you include a comparative analysis to highlight the uniqueness and validity of your results?

Thank you very much for your suggestions. We have included relevant content in the discussion section of our paper: In this study, we delved into the shared pathogenesis between PTC and RA, identifying key genes and their interconnected roles. Our findings offer novel insights and perspectives compared to existing research within this domain. Firstly, our identification of 64 DEGs that are common in both PTC and RA, along with our functional enrichment and PPI network analyses, provide a deeper understanding of their roles in immune response and signal transduction pathways. Notably, our emphasis on the potential synergistic roles of CXCL10 and IL6 in both diseases is a novel contribution that has not been extensively explored in previous studies. Secondly, our comprehensive bioinformatics approach, coupled with the validation of our findings through clinical specimens, strengthens the reliability of our results. This stands in contrast to some studies that rely on a single data source or lack clinical validation, offering a more robust and thorough investigation. Furthermore, our exploration of potential shared pathological pathways, such as the PPAR signaling pathway, the AMPK signaling pathway, and the adipocytokine signaling pathway, highlights the intricate interplay between metabolic and immune pathways in the pathogenesis of PTC and RA. This emphasis on the crosstalk between these pathways provides a new perspective for understanding the complex mechanisms underlying these diseases and could pave the way for the development of novel therapeutic strategies targeting these pathways. Lastly, the validation of our findings in independent datasets further substantiates the significance of the identified hub genes in PTC and RA. This reinforces the generalizability and clinical relevance of our conclusions, addressing concerns about the reproducibility of results that some studies may face. In summary, by uncovering the common pathological pathways and key genes between PTC and RA, our study not only contributes new perspectives to the field but also provides potential biomarkers for future diagnostics and therapeutics. We anticipate that these findings will stimulate further research into the interconnected mechanisms of these two diseases, ultimately leading to the development of more effective treatment strategies.

4. Could you elaborate on the potential clinical implications of your identified hub genes and pathways? How might these findings influence future research or therapeutic approaches?

Thank you very much for your suggestion. We have included relevant content regarding the clinical significance in the discussion section of our paper: The identification of hub genes and pathways in our study offers profound implications for the clinical management of PTC and RA, from diagnostics to therapeutics. The discovery of differentially expressed genes, such as CXCL10 and IL6, presents opportunities for developing diagnostic biomarkers that could indicate disease presence or severity, aiding in early detection and patient stratification. Moreover, these genes have the potential to act as prognostic indicators, providing insights into disease progression and patient outcomes, with elevated levels of CXCL10 in RA, for example, linked to osteoclast formation and disease severity. The central role of these genes in disease pathogenesis also positions them as potential therapeutic targets, where interventions such as small molecule inhibitors or monoclonal antibodies could disrupt inflammatory processes common to both diseases. Our findings further support the shift towards personalized medicine, where treatment plans could be tailored based on the expression levels of these genes, maximizing efficacy and minimizing side effects. The shared pathways between PTC and RA also open new avenues for research, inviting investigations into their mechanistic links and the exploration of these genes in other disease contexts. This understanding could inform the development of combination therapies targeting multiple aspects of the disease process, such as the simultaneous modulation of the AMPK and PPAR pathways. Additionally, the identified genes could serve as real-time monitors of treatment response, allowing for dynamic adjustments in patient care. In essence, our identification of shared DEGs not only deepens our comprehension of the molecular foundations of PTC and RA but also lays a groundwork for innovative diagnostic, prognostic, and therapeutic strategies that have the potential to revolutionize their clinical management.

5. The authors should discuss the topic as the future direction. Important literature in these fields should be cited. Some recommended studies might be helpful (PMIDs: 37008987, 33996830, 26295845, 33996831, 33114569, 24416258, 23481571).

Thank you for your reminder. we have included discussions on future research and cited relevant literature in our article: Our study opens several avenues for future research that could significantly enhance our understanding of PTC and RA. The identification of shared DEGs such as CXCL10 and IL6 invites further investigation into their mechanistic roles, building on studies that have underscored the importance of chemokines in immune regulation and cancer progression (PMID: 37008987). Future work could also focus on validating these genes as therapeutic targets, especially considering the impact of pathway targeting observed in recent research (PMID: 33996830, 26295845, 33996831). The development of personalized medicine approaches, informed by the genetic heterogeneity revealed in studies (PMID: 33114569), may lead to more tailored treatments. Additionally, exploring combination therapies that modulate multiple pathways simultaneously, as suggested by research into the synergistic effects of combined treatments (PMID: 24416258), could improve patient outcomes. Longitudinal studies that track the expression of these genes may offer insights into disease dynamics and treatment responses, as highlighted by the dynamic nature of gene expression in RA (PMID: 23481571). Lastly, the exploration of immune modulation therapies targeting the identified pathways could be a promising direction, inspired by research into the modulation of immune responses by cytokines and chemokines (PMID: 37008987). Collectively, these future research directions, supported by the referenced literature, hold great promise for advancing our knowledge and treatment strategies for PTC and RA.

Reviewer #2: 1.English expressions need to be edited more careful and more native, in this manuscript, there are many mistakes. The authors should pay attention to the problem of two ends aligned. For example, “These e” on the 4 line of Methods in the abstract.

Thank you very much for your suggestions. We have retouched the full text.

2. The workflow in Figure 1 is unspecific and less organized. It's more like a stack of terminologies than a higher-level summary of the existing data and methodologies, please improve it.

Thank you very much for your suggestions. We have redrafted Figure 1 and have simplified and summarized the content.

3. I suggest the authors should elaborate their motivation in the discussion section, as they use several common bioinformatics analysis methods and online tools. What is the novelty and technicality of their work?

Thank you for your reminder; we have incorporated the novelty of our research and other content you mentioned into our article: Our study's novelty emerges from the synergistic application of established bioinformatics methods to explore the shared pathogenesis of PTC and RA. While individually these methods are common, their integrated use provides a unique and comprehensive analysis that has not been previously employed in this context. By combining gene expression profiling, functional enrichment, PPI network analysis, and transcription factor validation, we have identified a core set of DEGs that are pivotal to both diseases. The technical strength of our work lies in the rigorous bioinformatics approach followed by empirical validation using clinical samples, ensuring the reliability and clinical relevance of our findings. Our use of advanced tools like DESeq2 for data normalization and TRRUST for regulatory network prediction has enabled a robust examination of the molecular interplay between PTC and RA. Moreover, our study introduces a systems biology perspective that surpasses traditional methodologies, offering a holistic view of the disease mechanisms and revealing potential therapeutic targets that may have been obscured by more focused analyses. In essence, the innovation of our research is not in the use of common methods, but in how we have harnessed them to offer new insights into PTC and RA, setting a new standard for integrative bioinformatics studies in this field.

4. Advancements in computational capabilities have led to the widespread application of machine learning techniques, especially deep learning, across various bioinformatics fields. Important computational models in these fields should be cited. Some recommended studies are helpful (PMIDs: 36305458, 34232474, 35817399, 37525507, 38040204, 38737196, 38274493, 38844122, and doi:10.1016/j.swevo.2024.101567).

Thank you for your suggestion; we have cited the corresponding literature in the eighth comment.

5. The authors should carefully check and unify the information of references. Some references lack the information of volume or contain the wrong page number.

Thank you for your suggestion; we have re-proofread the full text.

6. There should be a space between the reference and other word.

Thank you very much for your suggestion; It is reflected in the manuscript.

7. The authors claim that the expression of CXCL10 was confirmed in clinical specimens, highlighting its crucial function in both PTC and RA. I think more validation should be adopted. Especially, I want to see some associations can be confirmed by other method or biological experiments.

Thank you very much for your suggestion. We agree that more study would be useful to understand details of interaction and enhance. At the point, we do not have the necessary tool-set to study the problem. We will fully consider this view and put it into practice in future experiments to further explore and verify the deeper relationship between PTC and RA.

8. Future work and limitations of the proposed algorithm should be addressed detailly in the manuscript for further research.

While our study sheds new light on the shared pathogenesis of PTC and RA, it also underscores the imperative for sustained research efforts to transcend current methodological constraints and deepen our comprehension of these interconnected diseases. Our pioneering work in verifying common pathogenic genes, such as CXCL10, through retrospective clinical sample analysis, lays a groundwork for further exploration of the molecular mechanisms at play in PTC and RA. Nonetheless, we recognize the inherent limitations in our research, including the relatively constrained scope of clinical data for verification, which may influence the robustness of our conclusions, and the necessity for more exhaustive investigation into the functional pathways of genes like CXCL10. The evolution of computational power and the burgeoning application of machine learning, particularly deep learning, offer promising avenues for overcoming these limitations. By incorporating sophisticated computational models from the literature, including applications of machine learning, deep learning, and other advanced algorithms [62-70], the precision and profundity of bioinformatics analyses can be substantially augmented. These models can provide a more nuanced understanding of complex biological systems, potentially illuminating the intricate PPI network interactions and revealing novel biomarkers that our current datasets could not fully encapsulate. Future studies should leverage these computational advances to analyze larger and more diverse cohorts, leading to more generalized findings and possibly uncovering population-specific biomarkers. Incorporating multi-omics data, including proteomics and epigenetics, alongside machine learning algorithms could offer a comprehensive molecular landscape, facilitating the development of predictive models for personalized treatment strategies. Moreover, the dynamic progression of diseases calls for time-series analysis, which, when combined with machine learning, could offer insights into the temporal changes in disease pathology, guiding the creation of stage-specific therapeutic interventions. In conclusion, our study marks a notable step forward in understanding the shared pathogenesis of PTC and RA. By addressing the limitations of our methodologies and embracing the potential of emerging computational technologies, we are poised to expand our knowledge and bolster our clinical capabilities in managing these intricate diseases.
