# Supplementary material for: Identification and validation of the common pathogenesis and hub biomarkers in Papillary thyroid carcinoma complicated by rheumatoid arthritis
Source: PLoS One. 2025 Mar 10;20(3):e0317369. doi: 10.1371/journal.pone.0317369 (PMC11892850; doi:10.1371/journal.pone.0317369)
Supplement: Attachment — Submitted filename: response2.docx [file pone.0317369.s003.docx]

Dear reviewers, thank you for your careful review and constructive suggestions regarding our manuscript. We have revised the manuscript in accordance with the comments and marked all the amends on our revised manuscript. And point-by-point responses to the comments were as follows:

Reviewer #1: The authors have revised the manuscript accordingly. And now the manuscript seems acceptable for publication.

Thank you very much for recognizing our work.

Reviewer #2: The author has already addressed all of the issues. I think the manuscript can be published in PLOS ONE.

Thank you very much for recognizing our work.

Reviewer #3: Dear Editor

I read carefully the manuscript submitted your journal and it’s potential for publication after minor revision.

To improvement the manuscript suggested the usefulness resources in below.

https://doi.org/10.1002/jcb.28425

<https://doi.org/10.18502/jabs.v12i1.8872>

Thank you very much for your suggestions, we have learned and referenced both articles in the article.

Reviewer #4: I have carefully evaluated the revision work by authors. I am pleased to report that all concerns are addressed properly. However, I have some minor isssues for authors' reference.

1) The layout of the figures is really unsightly, especially for figure 3, 4,5,... Actually, each figure contains limited data and occupy large space. Please consider to merge some figures into one and improve the figure quality.

Thank you very much for your feedback. We have already merged Figure 4.5 and changed the resolution to 300 dpi.

2) Due to very limited experimental validations were conducted in this paper, please comprehensively specify all the potential limitations of extending current results into real-world clinical practice.

Thank you for your review of our article and the valuable comments provided. We acknowledge that there is indeed a limitation in the experimental validation part of our current study. Below is a comprehensive explanation of the limitations of our research and the potential impact these limitations may have on clinical practice:

Limitations in experimental design: Due to constraints in resources and time, our experimental design may not have covered all relevant clinical variables. In actual clinical settings, further research is needed to explore how these variables affect the occurrence and development of diseases.

Insufficient clinical sample validation: Although we have validated the expression of key genes such as CXCL10, these validations were conducted on a limited number of clinical samples. Before applying these findings to clinical practice, further validation in a broader patient population is required.

Lack of long-term follow-up: Our study lacks long-term tracking of patient outcomes, which is crucial for assessing the effectiveness of treatments and the progression of diseases. In clinical practice, long-term studies are necessary to monitor the effects of treatments and the quality of patients' survival.

Lack of intervention measures: Our study primarily focused on the identification of biomarkers without further exploring intervention measures based on these biomarkers. In clinical practice, more research is needed to develop and test potential therapeutic strategies targeting these biomarkers.

To address these limitations, we recommend that future research include larger multicenter clinical trials and long-term follow-up studies. Additionally, interdisciplinary collaboration will help develop new technologies and methods to enhance the depth and breadth of our research. We believe that through ongoing research and improvement, our findings can provide valuable insights for the clinical management of PTC and RA. Thank you again for your valuable comments.

3) Please consider to add a table to summarize the ongoing or completed clinical trials that are related to your study topic .

Thank you very much for your suggestion. Given that our study is presently at its nascent phase, further investigation will be required in the future to translate our findings into clinical research applications. Due to the small number of relevant clinical trials, we summarized some of the literature and put them into the article as a table. The table has been included in the supplementary file.

4) Please consider to add a figure to describe the mechanistic pathway or molecular process of pathogenesis and hub biomarkers in Papillary thyroid carcinoma complicated with Rheumatoid arthritis.

Thank you very much for your proposal, but our research merely reveals a potential common hub biomarker. We have not yet further studied its downstream mechanisms or validated its clinical applications. Therefore, the pathogenic mechanisms and molecular processes of the two diseases are currently unclear. We will conduct further research in this area to fully explore and reveal its downstream mechanisms.

Reviewer #5: More molecular experiments should be added to further bolster the scientific rigor of this paper. At least, western blot and PCR... or other basic experiments should be performed to rich the mechanism contents.

Thank you very much for your suggestion; we have included QPCR and WB experiments in the article for further validation.
